# Oleanane triterpenoid CDDO-Me induces apoptosis in multidrug resistant osteosarcoma cells through inhibition of Stat3 pathway

**DOI:** 10.1186/1471-2407-10-187

**Published:** 2010-05-10

**Authors:** Keinosuke Ryu, Michiro Susa, Edwin Choy, Cao Yang, Francis J Hornicek, Henry J Mankin, Zhenfeng Duan

**Affiliations:** 1 Department of Orthopaedic Surgery, Massachusetts General Hospital, Boston, MA 02114, USA; 2 Sarcoma Biology Laboratory, Center for Sarcoma and Connective Tissue Oncology, Massachusetts General Hospital, Boston, MA 02114, USA; 3 Department of Orthopaedic Surgery, Nihon University School of Medicine, Tokyo 160-0015, Japan

## Abstract

**Background:**

The activation of signal transducer and activator of transcription 3 (Stat3) pathway correlates with tumor growth, survival, drug resistance and poor prognosis in osteosarcoma. To explore the potential therapeutic values of this pathway, we assessed both the expression and the activation of Stat3 pathway in several pairs of multidrug resistant (MDR) osteosarcoma cell lines, and tissues. To explore the potential therapeutic values of this pathway, we analyzed the ability of the synthetic oleanane triterpenoid, C-28 methyl ester of 2-cyano-3,12-dioxoolen-1,9-dien-28-oic acid (CDDO-Me), to inhibit Stat3 expression and activation as well as its effects on doxorubicin sensitivity in osteosarcoma cells.

**Methods:**

Expression of Stat3, phosphorylated Stat3 (pStat3) and Stat3 targeted proteins, including Bcl-X_L_, Survivin and MCL-1 were determined in drug sensitive and MDR osteosarcoma cell lines and tissues by Western blot analysis. The effect of CDDO-Me on osteosarcoma cell growth was evaluated by MTT and apoptosis by PARP cleavage assay and caspase-3/7 activity.

**Results:**

Stat3 pathway was activated in osteosarcoma tissues and in MDR cell lines. CDDO-Me inhibited growth and induced apoptosis in osteosarcoma cell lines. Treatment with CDDO-Me significantly decreased the level of nuclear translocation and phosphorylation of Stat3. The inhibition of Stat3 pathway correlated with the suppression of the anti-apoptotic Stat3 targeted genes Bcl-X_L_, survivin, and MCL-1. Furthermore, CDDO-Me increased the cytotoxic effects of doxorubicin in the MDR osteosarcoma cell lines.

**Conclusions:**

Stat3 pathway is overexpressed in MDR osteosarcoma cells. CDDO-Me significantly inhibited Stat3 phosphorylation, Stat3 nuclear translocation and induced apoptosis in osteosarcoma. This study provides the framework for the clinical evaluation of CDDO-Me, either as monotherapy or perhaps even more effectively in combination with doxorubicin to treat osteosarcoma and overcome drug resistance.

## Background

Osteosarcoma is the most common malignant tumor of bone, which mainly affects children and adolescents [1,2]. Current treatment of osteosarcoma consists of multi agent chemotherapy and surgical resection[3]. The advancement in intensive chemotherapy has significantly improved the 5-year survival rate from 10% with surgery alone to approximately 60-70% when combined with chemotherapy [1-4]. Even so, for 20 years, survival rate has not changed and nearly 30-40% of the patients still experience local recurrence or metastasis, possibly because of development of multidrug resistance (MDR). Overcoming drug resistance is one approach to improve the survival rate of osteosarcoma patients. The development of drug resistance is associated with many events, such as activation of transcription factors, overexpression of antiapoptotic proteins, and overexpression of multidrug resistance gene 1 (MDR1) [5-8]. Successful management of osteosarcoma might be greatly aided by the use of novel agents that could overcome drug resistance.

Signal Transducer and Activator of Transcription 3 (Stat3) is one of the transcription factors that play an important role in tumor cell growth, survival, proliferation, differentiation, apoptosis, metastasis, angiogenesis and drug resistance [9-17]. Stat3 is activated (phosphorylated) by Janus-activated kinase (JAK)-1 or JAK-2 in response to interleukin-6 (IL-6) family of cytokines and growth factors [18]. Stat3 then forms homodimers that translocate to the cell nucleus and binds to promoters of target genes, activating oncogenes such as c-myc and cyclin D, and antiapoptotic proteins [19]. Constitutive activation of Stat3 pathway has been found in many cancer cells including osteosarcoma [9-11,13,14,16,17,20]. Furthermore, constitutively activated Stat3 pathway correlates with malignant tumor phenotype, resistance to chemotherapeutic drugs, and poor prognosis in some cancers [8,15,17,21-27].

Several reports have shown inhibition of Stat3 pathways in cancer cells, resulting in a dramatic increase of apoptosis [13,17,20,28-32]. The novel synthetic oleanane triterpenoid, C-28 methyl ester of 2-cyano-3,12-dioxoolen-1,9-dien-28-oic acid (CDDO-Me), is a promising new class of agents for the prevention and treatment of cancer [33-35]. When CDDO-Me is applied at low concentrations, it demonstrated a variety of anti-inflammatory effects. At higher concentrations, the compound inhibits cancer cell growth and proliferation in a wide variety of cell lines including ovarian, cervical, breast, liver, leukemia, and lung cancer [17,32-38]. CDDO-Me is currently in phase I/II clinical trials for cancer treatment [36-39]. CDDO-Me-induced apoptosis is associated with the activation of caspase 3 and 8, cytochrome c, SOCS-1, and SHP-1, and inhibition of NF-kB, Cox2 and VEGF [32,33,35,40]. To date, the effect of CDDO-Me on MDR osteosarcoma cells is unclear.

In this study, we investigated the molecular mechanism of CDDO-Me induced apoptosis and the effects of combinations of CDDO-Me with doxorubicin which is known to have established activity in the clinical therapy for osteosarcoma.

## Methods

### Cell lines, tissues, antibodies and drugs

Human osteosarcoma cells KHOS, U-2OS, SaOS were obtained from the American Type Tissue Collection (Rockville, MD) and a normal human hipbone osteoblast cell line HOB-c was obtained from the Promo Cell GmbH (Heidelberg, Germany). The MDR cell line U-2OS_TR _was established as previously reported [1,2]. Briefly, the U-2OS cell lines were selected over a period of 8 months by continuous culturing in medium containing step-wise increases in drugs. Dr. Efstathios Gonos (Institute of Biological Research and Biotechnology, Athens, Greece) kindly provided the MDR KHOS_R2 _cell line [41]. In addition, osteosarcoma tissue samples were obtained from the Massachusetts General Hospital Sarcoma Tissue Bank. Surgically treated patients diagnosed with osteosarcoma were identified and utilized for the study and were used in accordance with the policies of the Institutional Review Board of the institution. Doxorubicin was obtained through unused residual clinical material at the Massachusetts General Hospital. CDDO-Me was kindly provided by Dr. Jeff Supko (Massachusetts General Hospital). The stock solution of drugs were prepared according to the drug specifications and stored at -20°C. The rabbit polyclonal antibodies to Stat3, Bcl-X_L_, MCL-1, PARP, and the mouse monoclonal antibodies to phosphorylated Stat3 (pStat3), survivin were purchased from Cell Signaling Technologies (Cambridge, MA). The mouse monoclonal antibody to β-actin and MTT reagent were purchased from Sigma-Aldrich (St. Louis, MO). The Pgp1 monoclonal antibody C219 was purchased from Signet (Dedham, MA). Goat antimouse-HRP and goat antirabbit-HRP were purchased from BIO-Rad (Hercules, CA). SuperSignal^® ^West Pico Chemiluminescent Substrate was purchased from PIERCE (Rockford, IL).

### Cell culture

Osteoblast cells HOB-c were cultured in Osteoblast Growth Medium (Promo Cell), and all other cell lines were cultured in RPMI 1640 supplemented with 10% fetal bovine serum, 100 U/ml penicillin, and 100 μg/ml streptomycin (all obtained from Invitrogen, Carlsbad, CA). All cells were incubated at 37°C in 5% CO_2_-95% air atmosphere and passaged when near confluent monolayers were achieved using trypsin-EDTA solution. Resistant cell lines were continuously cultured in 0.1 μM doxorubicin. Cells were free of mycoplasma contamination as tested by MycoAlert^® ^Mycoplasma Detection Kit from Cambrex (Rockland, ME).

### Western blotting

Protein lysates from osteosarcoma cells and tissues were generated through lysis with 1× radioimmunoprecipitation assay lysis buffer (Upstate Biotechnology, Charlottesville, VA). The concentration of the protein was analyzed by DC Protein assay (Bio-Rad Laboratories, Hercules, CA) and spectrophotometer (Beckman DU-640, Beckman Instruments, Inc., Columbia, MD). Thirty micrograms of total protein was processed on Nu-Page 4-12% Bis-Tris Gel (Invitrogen) and transferred to a pure nitrocellulose membrane (Bio-Rad) then immunoblotted with specific antibodies. Primary antibodies were incubated at 1:1000 dilution in Tris-buffered saline, pH 7.4, with 0.1% Tween-20 and 5% nonfat milk (Bio-Rad) overnight at 4°C. Horseradish peroxidase-conjugated secondary antibodies (Bio-Rad) were incubated in Tris-buffered saline, pH 7.4, with 0.1% Tween-20 and 5% nonfat milk, at 1:2,000 dilutions for 1 hour at room temperature. Positive immunoreactions were detected by using SuperSingal West Pico Chemiluminescent Substrate (Pierce, Rockford, IL).

### Cell viability analysis

The In vitro cell viability analysis was performed by MTT assay as described previously [42]. For analyzing effects of CDDO-Me on KHOS, KHOS_R2_, U-2OS, U-2OS_TR _and HOB-c, 2 × 10^3 ^cells per well were plated in 96-well plates in culture medium containing increasing concentrations of CDDO-Me. For the effect of varying concentrations of CDDO-Me on U-2OS_TR _and KHOS_R2_, 2 × 10^3 ^cells per well were plated in 96-well plates in culture medium containing increasing concentrations of doxorubicin and CDDO-Me at final concentrations of 0.1 and 0.3 μmol/L, respectively. After 7 days of culture at 37°C, 10 μl MTT (5 mg/ml in PBS, obtained from Sigma) was added to each well and the plates were incubated for 3 h. The resulting formazan product was dissolved with acid-iso-propanol and the absorbance at a wavelength of 490 nm (A_490_) was read on a SPECTRAmax Microplate Spectrophotometer (Molecular Devices, Sunnyvale, CA). The absorbance values were normalized by assigning the value of the control line in the medium without drug to 1.0 and the value of the no cell control to 0. Experiments were performed in triplicate. The half inhibitory concentration (IC_50_) was defined as the compound or chemo drug concentration required decreasing the A_490 _to 50% of the control value.

### Apoptosis assay

Lysates from whole-cell were immunoblotted with specific antibodies to PARP (Cell Signaling Technology) and its cleavage products. Positive immunoreactions were detected by using Super Signal West Pico Chemiluminescent Substrate. As a second parameter of apoptotic cell death, we measured caspase-3/7 activities in HOB-c, KHOS and KHOS_R2 _after treatment with CDDO-Me by using Apo-ONE Homogenous caspase-3/7 system according to the manufacturer's instructions (Promega, Madison, WI). The intensity of the emitted fluorescence was determined at a wavelength of 521 nm with the use of a SPECTRAmax^® ^Microplate Spectrofluorometer (Molecular Devices).

### Real-time analysis of the effect of CDDO-Me on Stat3 nucleocytoplasmic translocation

To study the effects of CDDO-Me on Stat3 nuclear translocation in live cells, a real-time cell based assay was used as described below. Stable transfectants expressing EGFP-Stat3 were generated with the osteosarcoma cell line U-2OS using standard lipofectmine transfection techniques with G418 selection. EGFP-Stat3 expressing cells were seeded at a density of 4 × 10^3 ^cells per well in 96 well plates and incubated overnight at 37°C. The cells were then treated with either 1 μM CDDO-Me with 30 ng/ml human recombinant IL-6 (R&D Systems, Minneapolis, MN) or IL-6 alone. To counterstain the nuclei, the cells were incubated with 1 μg/ml Hoechst 33342 (Invitrogen, Carlsbad, CA) for 1 minute. IL-6 dependent nuclear translocation of EFGP-Stat3 was analyzed using an Olympus 1X71 fluorescence microscope and the pictures were captured as digital images using IPLab Software from Scanalytics (Rockville, MD).

### Data analysis

Values shown are representative of triplicate determinations in two or more experiments. The IC_50 _was calculated and the effects of treatment were evaluated using a two-sided Student's *t *test (GraphPad PRISM^® ^4 software, GraphPad Software, San Diego, CA). Errors are a SD (standard deviation) of averaged results and with value *p *< 0.05 was considered as a significant difference between means.

The combination index (CI) for experimental treatment combinations was calculated to determine the synergistic, additive, or antagonistic effects using the Chou-Talalay method and GraphPad PRISM^® ^4 software[43]. When CI = 1, the equation represents the conservation isobologram and indicates additive effects. CI values of <1.0 indicates synergistic effect, > 1.0 indicates antagonistic effect.

## Results

### Stat3 pathway is activated in osteosarcoma cell lines and tissues

**Figure 1:**
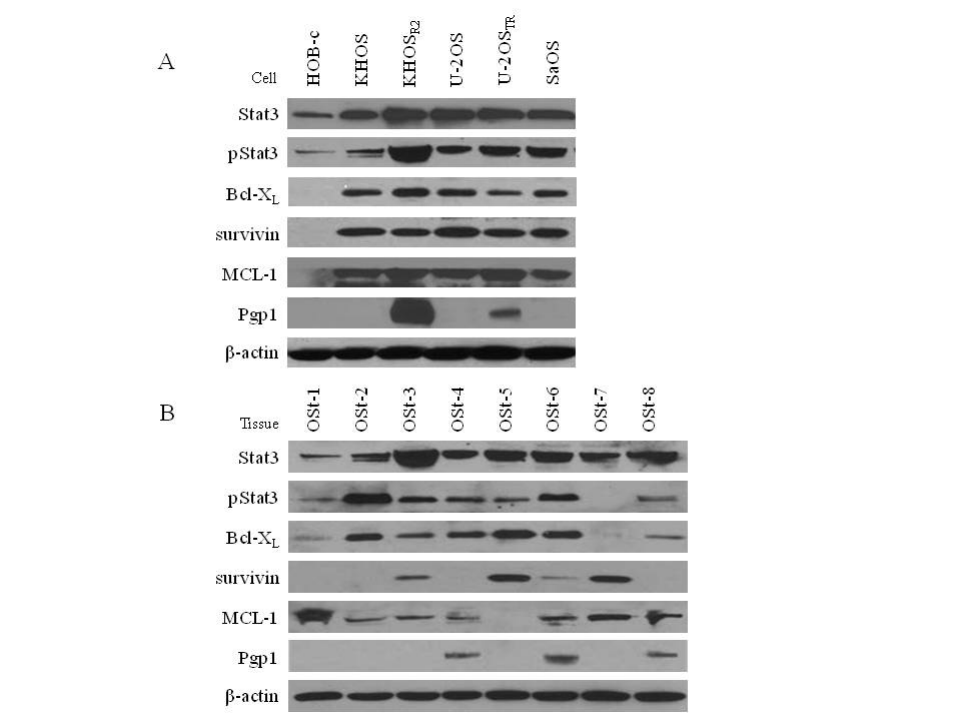
**Activation of Stat3, pStat3, Stat3-mediated antiapoptotic proteins, and Pgp1 in osteosarcoma cell lines and tissues**. A) Osteoblast cell lines, drug sensitive and MDR osteosarcoma cell lines. B) Osteosarcoma tissues. Expression was assessed with total cellular protein isolated from the indicated cell lines and immunoblotted with specific antibodies as described in Materials and Methods. The blots were also probed with an anti-actin monoclonal antibody to assess relative protein levels in the sample lanes.

To evaluate the expression and activation of Stat3 pathway, we analyzed the protein expression in several pairs of both drug sensitive and MDR osteosarcoma cell lines and 8 samples of osteosarcoma tissues. Western blot analysis demonstrated that Stat3 and pStat3 were constitutively overexpressed in drug sensitive cell lines KHOS, U-2OS, SaOS and MDR cell lines KHOS_R2_, U-2OS_TR_. Normal osteoblast cell line HOB-c expressed Stat3 as well as low levels of pStat3 (Fig. 1A). In osteosarcoma tissues, Stat3 was overexpressed in all of the samples and pStat3 was overexpressed in 7 out of 8 (88%) samples (Fig. 1B). Following this preliminary study, we analyzed the expression of Stat3-mediated antiapoptotic proteins Bcl-X_L_, survivin, and MCL-1. Western blot analysis demonstrated that Bcl-X_L_, survivin, and MCL-1 were constitutively overexpressed in drug sensitive cell lines and MDR osteosarcoma cell lines. Osteoblast cell lines HOB-c controls showed no expression of Stat3-mediated anti-apoptotic proteins. Pgp1 is also over expressed in MDR cell lines (Fig. 1A). Osteosarcoma tissues showed heterogeneous expression of Stat3 mediated antiapoptotic proteins and Pgp1 (Fig. 1B).

### CDDO-Me inhibits growth and induces apoptosis in osteosarcoma cells

**Figure 2:**
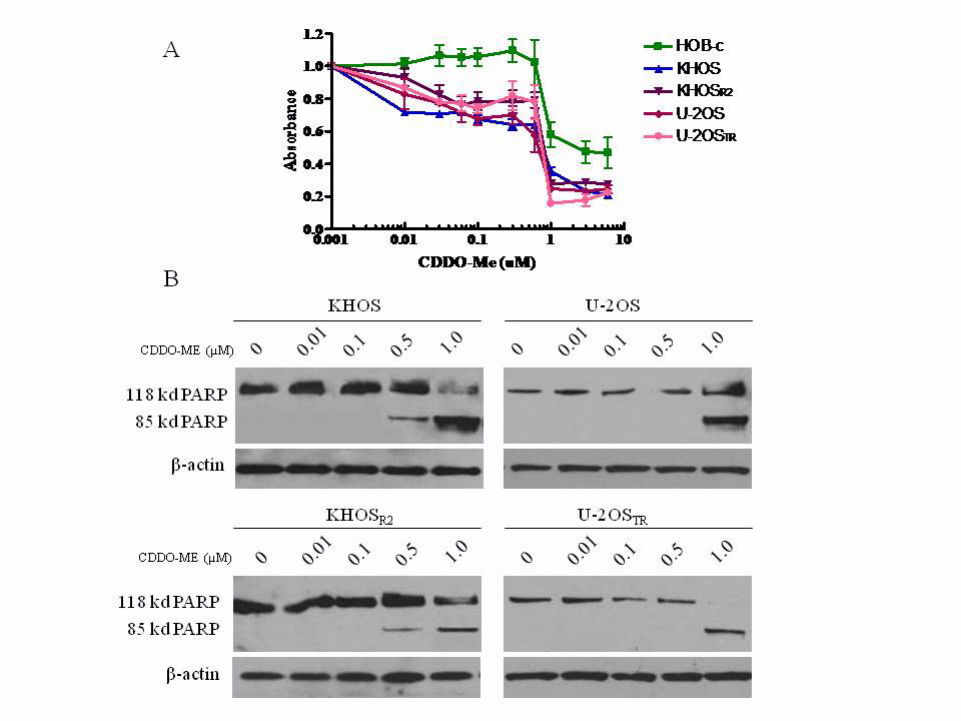
**CDDO-Me inhibits growth and induces apoptosis in osteosarcoma cell lines**. A) To analyze the effect of CDDO-Me, KHOS, U-2OS, KHOS_R2_, U-2OS_TR_, and HOB-c were exposed to varying concentrations of CDDO-Me for 7 days. Growth inhibition was assessed by MTT. B) Parental cells KHOS, U-2OS and MDR cells KHOS_R2 _and U-2OS_TR _were treated with CDDO-Me in a dose-dependent manner. Total cellular proteins were subjected to immunoblotting with specific antibodies to PARP and β-actin. PARP cleavage was detected in all four osteosarcoma cell lines after treatment with CDDO-Me. The anti-PARP antibodies demonstrate 118 kd full length PARP and 85 kd cleaved PARP fragment.

**Figure 3:**
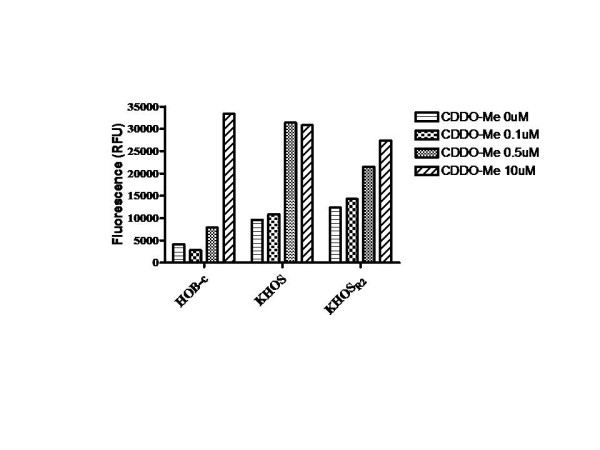
**Caspase-3/7 activity was measured as a second parameter of apoptotic cell death**. KHOS and KHOS_R2 _showed significant increase in apoptosis when they were treated with increasing concentrations of CDDO-Me. The experiment was repeated three times in triplicate.

To confirm that CDDO-Me inhibits cell growth, KHOS, U-2OS, KHOS_R2_, U-2OS_TR_, and HOB-c were evaluated using MTT assay. The results showed that the growth of all cell lines was inhibited after treatment with CDDO-Me (Fig. 2A). CDDO-Me showed significantly higher antiproliferative activity in osteosarcoma cells than in osteoblast cells (*p *< 0.0001). The IC_50 _of each cells were HOB-c: 0.8 μM, KHOS: 0.15 μM, KHOS_R2_: 0.33 μM, U-2OS: 0.17, U-2OS_TR_: 0.39 μM. The effect of CDDO-Me on the induction of apoptosis was assessed by evaluating PARP cleavage and caspase assay for both drug sensitive and MDR osteosarcoma cell lines. PARP cleavage was detected in all cells after 24 hours of treatment with CDDO-Me. A dose-response analysis revealed the appearance of PARP cleavage products in the presence of 0.5 μmol/L of CDDO-Me for KHOS, KHOS_R2 _and 1.0 μmol/L of CDDO-Me for U-2OS, U-2OS_TR _(Fig. 2B). In addition, caspase-3/7 activity was significantly increased when KHOS and KHOS_R2 _were treated with increasing concentration of CDDO-Me (Fig. 3).

### CDDO-Me inhibits IL-6 induced nuclear translocation of Stat3

**Figure 4:**
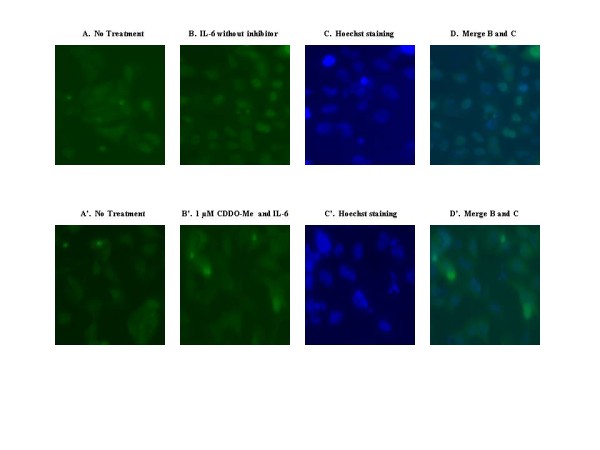
**CDDO-Me inhibits EGFP-Stat3 nuclear translocation in osteosarcoma cell line U-2OS**. U-2OS cells which stably express the EGFP-Stat3 fusion protein were incubated for 4 h with either IL-6 alone (A-D) or CDDO-Me (1 μM) followed immediately thereafter with the addition of IL-6 to a final concentration of 30 ng/ml (A'-D'). To counterstain the nuclei, the cells were incubated with 1 μg/ml Hoechst 33342 (Invitrogen, Carlsbad, CA) for 1 minute. Cells were photographed 1 h later. Subcellular localization of the fusion protein was assessed by fluorescence microscopy.

To identify the interruption of IL-6 dependent Stat3 nuclear translocation by CDDO-Me, a novel real-time cell-based method was developed to image the EGFP-Stat3 chimera in the nucleus and cytoplasm in human osteosarcoma cell line U-2OS. Resting cells demonstrated that the majority of EGFP-Stat3 was cytoplasmic (Fig. 4A, A') until the addition of IL-6, which then promptly induced translocation of fluorescent Stat3 to the nucleus in U-2OS cells (Fig. 4B). Pretreatment of the cells with CDDO-Me (1 μM) blocked IL-6 dependent EGFP-Stat3 nuclear translocation (Fig. 4B').

### CDDO-Me inhibits Stat3 pathway in a dose-dependent manner

**Figure 5:**
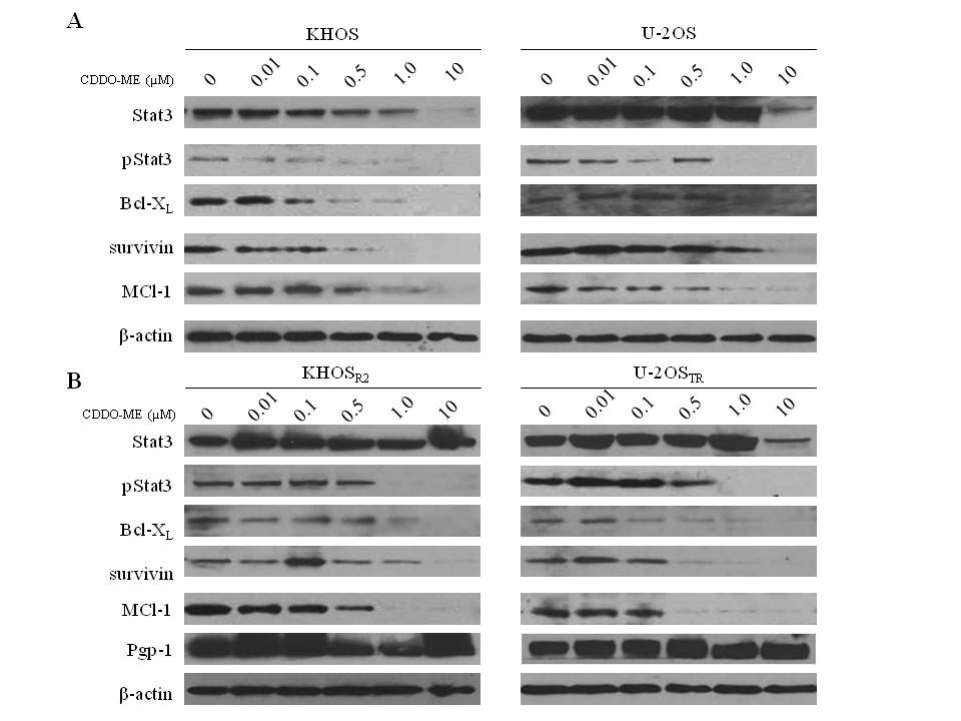
**Dose-dependent inhibition of Stat3 phosphorylation and down-regulated Stat3-mediated antiapoptotic proteins by CDDO-Me**. Parental cells KHOS, U-2OS (A) and MDR cells KHOS_R2_, U-2OS_TR _(B) were treated with CDDDO-Me for 24 hours in and then harvested and processed for Western blotting. For Western blot analysis, 30 μg of total cellular proteins were subjected to immunoblotting with specific antibodies.

After identifying CDDO-Me as an inhibitor of Stat3 nuclear translocation in osteosarcoma cells, the effect of CDDO-Me on Stat3 phosphorylation was examined in osteosarcoma drug sensitive and MDR cell lines. To evaluate the dose-dependent inhibition of Stat3 activation, the cell lines were treated with CDDO-Me alternatively with varying doses for 24 h. In a dose-dependent manner, concentrations as low as 0.5 μM CDDO-Me inhibited Stat3 phosphorylation (Fig. 5). Stat3 phosphorylation and nuclear translocation are required for Stat3 transcriptional activity. We hypothesized that inhibition of nuclear transport should suppress transcription and subsequent translation of Stat3-mediated proteins. Therefore, we next examined whether exposure of cell lines to CDDO-Me resulted in decreased expression of the antiapoptotic proteins Bcl-X_L_, survivin and MCL-1 by dose-dependent inhibition (Fig. 5). Results showed incubation of CDDO-Me for 24 h caused significant down-regulated Bcl-X_L_, survivin and MCL-1 expression in both drug sensitive (Fig. 5A) and MDR (Fig. 5B) cell lines in a dose-dependent manner.

### CDDO-Me inhibits Stat3 pathway and induces apoptosis in a time-dependent manner

**Figure 6:**
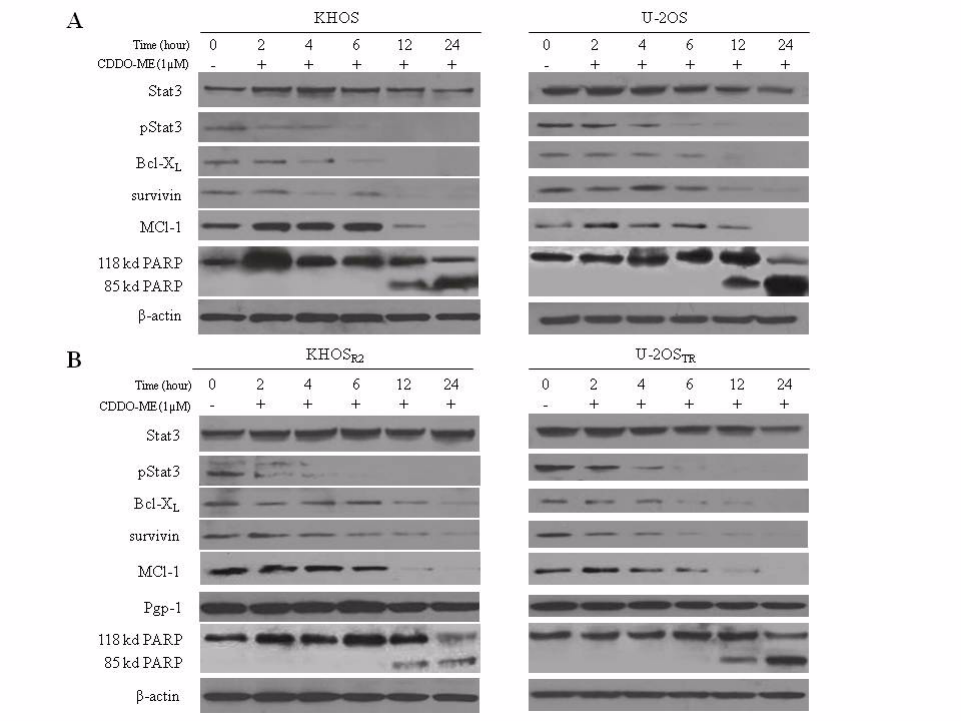
**Time-dependent inhibition of Stat3 phosphorylation and down-regulated Stat3-mediated antiapoptotic proteins by CDDO-Me**. Parental cells KHOS, U-2OS (A) and MDR cells KHOS_R2_, U-2OS_TR _(B) were treated for 24 h with CDDO-Me (1 μM), and then harvested and processed for Western blotting. For Western blot analysis, 30 μg of total cellular proteins were subjected to immunoblotting with specific antibodies.

To evaluate the time-dependent inhibition of Stat3 activation, the cell lines were treated with 1 μM of CDDO-Me for varying time periods for 24 h. In a time-dependent manner, the pStat3 level decreased as early as 4 hours after the addition of 1 μM of CDDO-Me treatment (Fig. 6). We next examined whether exposure of cell lines to CDDO-Me resulted in decreased expression of the antiapoptotic proteins Bcl-X_L_, survivin and MCL-1 by time-dependent inhibition (Fig. 6). Results showed incubation of CDDO-Me for 24 h caused significant down-regulation of Bcl-X_L_, survivin and MCL-1 expression in both drug sensitive (Fig. 6A) and MDR (Fig. 6B) cell lines in a time dependent manner. The effect of CDDO-Me on the induction of apoptosis was assessed by evaluating PARP cleavage in a time-dependent manner. PARP cleavage was detected in all cells after 12 hours of treatment with 1 μM of CDDO-Me (Fig. 6).

### CDDO-Me has no effect on Pgp-1 expression

While CDDO-Me significantly inhibits Stat3 phosphorylation in KHOS_R2_, U-2OS_TR_cells, there was no significant change in Pgp1 expression as observed in the Pgp1-over expressing cell lines (Fig. 5B and 6B).

### CDDO-Me shows synergy with doxorubicin and reduces resistance in MDR osteosarcoma cell lines

**Figure 7:**
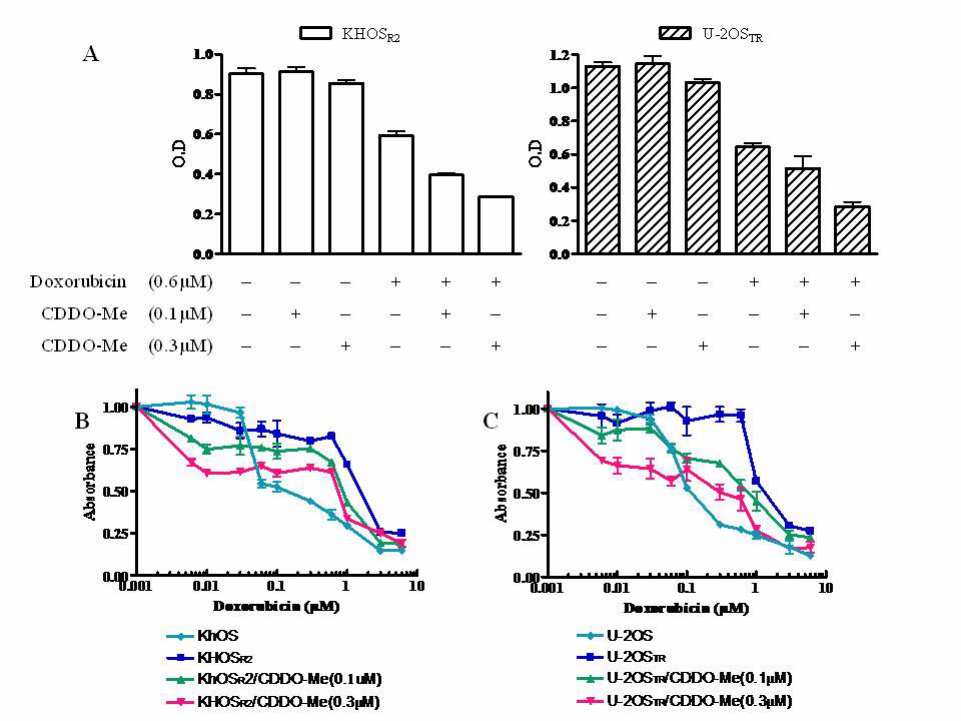
**CDDO-Me shows synergy with doxorubicin and overcomes drug resistance in MDR osteosarcoma cell lines**. A) CDDO-Me inhibits cell growth in KHOS_R2 _and U-2OS_TR_. Cells were seeded at a density of 8,000 cells per well in a 96-well plate for 24 hours. The cells were then treated with 0.6 μM doxorubicin, 0.1 μM CDDO-Me, 0.3 μM CDDO-Me or combination of different two drugs for additional 24 hours. The cells were analyzed by MTT assay as described in Materials and Methods (B and C). CDDO-Me together with doxorubicin overcome drug resistance in both MDR KHOS_R2 _and U-2OS_TR _cells.

To examine whether interruption of the Stat3 pathway shows synergistic effect when combined with chemotherapy, we analyzed the effect of CDDO-Me in the presence or absence of doxorubicin. Treatment of MDR cell lines with doxorubicin, CDDO-Me or combination of both drugs were analyzed using MTT. The combination of the two agents doxorubicin and CDDO-Me resulted in significantly greater cell death than either drug alone (Fig 7A). The combination index (CI) values were <1 in all cases and thereby identified synergistic interaction at all combinations between CDDO-Me and doxorubicin at various combination. The CI of each combinations were KHOS_R2_/doxorubicin+CDDO-Me(0.1 μM): 0.98, KHOS_R2_/doxorubicin+CDDO-Me(0.3 μM): 0.64, U-2OS_TR_/doxorubicin+CDDO-Me(0.1 μM): 0.83, U-2OS_TR_/doxorubicin+CDDO-Me(0.3 μM): 0.51.

To investigate the inhibition of Stat3 pathway's effect on drug sensitive osteosarcoma cell lines KHOS, U-2OS and MDR osteosarcoma cell lines KHOS_R2_, U-2OS_TR_, they were exposed to varying concentrations of doxorubicin and CDDO-Me for 7 days. MTT demonstrated that CDDO-Me has a synergistic effect on doxorubicin-induced antiproliferative effect in KHOS_R2 _and U-2OS_TR _(Fig 7B and 7C). In addition, MTT assay demonstrated that CDDO-Me increases doxorubicin's effect and partially overcomes doxorubicin-resistance. The IC_50 _of each cells were KHOS: 0.07 μM, KHOS_R2_/CDDO-Me(0.3 μM): 0.11 μM, KHOS_R2_/CDDO-Me(0.1 μM): 0.29 μM, KHOS_R2_: 0.86 μM (Fig 6B), and U-2OS: 0.06 μM, U-2OS_TR_/CDDO-Me(0.3 μM) 0.12 μM, U-2OS_TR_/CDDO-Me(0.1 μM): 0.24 μM, U-2OS_TR_: 0.93 μM (Fig 7C).

## Discussion

Stat3 pathway plays an important role in tumor cell growth and proliferation, and is constantly activated in many human cancer cell lines and tumor tissues [9-14,17,20]. Constitutive activation of Stat3 pathway could be an early indicator of drug resistance[15]. Activation of Stat3 pathway was also reported to be present in osteosarcoma cells and tissues [14,20]. In this study, we observed that elevated levels of Stat3 and pStat3 are detected in osteosarcoma drug sensitive cell lines KHOS, U-2OS, SaOS and osteosarcoma MDR cell lines KHOS_R2_, U-2OS_TR_. The data is consistent with previous studies that Stat3 pathways play an important role in not only drug sensitive but also drug resistant osteosarcoma cells [14,20]. The downstream effect of Stat3 activation is the Stat3-dependent regulation of several antiapoptotic genes including Bcl-X_L_, survivin, and MCL-1. Overexpressions of these survival-promoting genes have been shown to be highly expressed and prevent apoptosis in human cancer cells, especially in high-grade tumors [13,44-46]. In our study, these antiapoptotic genes were highly expressed in both drug sensitive and resistant osteosarcoma cell lines, but not in normal human osteoblast cells.

Nuclear translocation of pStat3 is a crucial event for its transcriptional function. Blocking the phosphorylation and translocation of Stat3 is a rational approach for the inhibition of Stat3 activation. Recently, Stat3 has been implicated as a promising target for cancer therapy [12,13,20,28-31]. Stat3 inhibitors, SD-1029 and SD-1008, greatly induce apoptosis in drug resistant ovarian cancer cells by blocking Stat3 nuclear translocation [29,30]. Stat3, pStat3 and Stat3 targeted antiapoptotic proteins are over expressed in drug resistance osteosarcoma cells. Inhibition of the Stat3 pathway and interruption of antiapoptotic response may also play an important role for treatment of these cells. Our result is consistent with recent reports showing that the novel Stat3 inhibitor, Indirubin, significantly induces apoptosis in human breast cancer cells [13]. In addition, LLL3, Stat3 DNA binding and transcription activities inhibitor, and Stat3 siRNA significantly decrease cell proliferation and viability, ultimately inducing apoptosis in osteosarcoma cells[20].

CDDO is widely used in Asian herbal medicine and was originally identified as an active compound for anti-inflammatory and anti-carcinogenic treatments [34,39]. The novel compound CDDO-Me has been shown to be highly effective in vivo models for the prevention and treatment of cancer [33-35]. CDDO-Me is able to induce the differentiation of tumor cells, suppress the growth of tumor cells, and induce apoptosis in cancer cells that are resistant to conventional chemotherapeutic agents [32]. Recent report has shown that CDDO-Me inhibits activation of the JAK/Stat3 pathway by forming adducts with both JAK1 and Stat3 in human cervical and breast cancer cells [34]. Previously, we also demonstrated that CDDO-Me inhibits Stat3 pathway in ovarian cancer cells by down-regulation of antiapoptotic genetic expression, and resulted in a dramatic induction of apoptosis [17]. Furthermore, recent study showed that the novel Stat3 target gene Bcl-X_L _inhibitor, ABT-737, greatly enhanced the activities of paclitaxel in lung cancer cells [47]. In this study, CDDO-Me inhibits Stat3 phosphorylation and down-regulates Stat3-mediated antiapoptotic proteins Bcl-X_L_, survivin and, MCL-1, in both dose and time dependent manner. This was through apoptotic cell death in both drug sensitive and resistant osteosarcoma cells. Furthermore, the apoptotic threshold also increases the sensitivity of these cells to the cytotoxic effects of doxorubicin. The results are consistent with a series of studies showing that CDDO-related compounds are not monofunctional drugs that strictly target single steps in signal transduction pathways [32,40,48]. Biochemical and cellular assays support the hypothesis that the molecule CDDO-Me inhibits Stat3 activity with resultant inhibition of Stat3 phosphorylation, nuclear translocation, and decrease in Stat3-dependent transcription, leading to apoptosis and enhanced chemosensitivity.

## Conclusion

Our results demonstrated that Stat3 pathways were constantly activated in osteosarcoma and MDR osteosarcoma cells. CDDO-Me significantly inhibits Stat3 phosphorylation and consequently Stat3 nuclear translocation, and eventually resulted in inducing apoptosis in these cells. In addition, this compound is able to overcome drug resistance in these cells.

## Competing interests

The authors declare that they have no competing interests.

## Authors' contributions

KR designed and carried out all the studies, analyzed the data, and drafted the manuscript. MS helped in carrying out the experiments and assisted with the manuscript draft. EC provided guidance with the study and assisted with the manuscript draft. CY helped in carrying out the experiments. FJH and HJM provided experimental reagents, osteosarcoma tissues and guided the team in the analysis of results and discussions. ZD is the principal investigator who led the research effort, provided guidance with the studies, assisted in data analysis and interpretation, and edited the manuscript. All authors read and approved the final manuscript.

## Pre-publication history

The pre-publication history for this paper can be accessed here:

http://www.biomedcentral.com/1471-2407/10/187/prepub

## References

[B1] Arndt CA Crist WM Common musculoskeletal tumors of childhood and adolescence N Engl J Med 1999 341 5 342 352 10.1056/NEJM199907293410507 10423470

[B2] Mirabello L Troisi RJ Savage SA Osteosarcoma incidence and survival rates from 1973 to 2004: data from the Surveillance, Epidemiology, and End Results Program Cancer 2009 115 7 1531 43 10.1002/cncr.24121 19197972 PMC2813207

[B3] Ferrari S Palmerini E Adjuvant and neoadjuvant combination chemotherapy for osteogenic sarcoma Curr Opin Oncol 2007 19 4 341 346 10.1097/CCO.0b013e328122d73f 17545797

[B4] Bacci G Ferrari S Bertoni F Ruggieri P Picci P Longhi A Casadei R Fabbri N Forni C Versari M Long-term outcome for patients with nonmetastatic osteosarcoma of the extremity treated at the istituto ortopedico rizzoli according to the istituto ortopedico rizzoli/osteosarcoma-2 protocol: an updated report J Clin Oncol 2000 18 24 4016 4027 11118462 10.1200/JCO.2000.18.24.4016

[B5] Yang C Yang S Wood KB Hornicek FJ Schwab JH Fondren G Mankin H Duan Z Multidrug resistant osteosarcoma cell lines exhibit deficiency of GADD45alpha expression Apoptosis 2009 14 1 124 133 10.1007/s10495-008-0282-x 19052873

[B6] Orr GA Verdier-Pinard P McDaid H Horwitz SB Mechanisms of Taxol resistance related to microtubules Oncogene 2003 22 47 7280 7295 10.1038/sj.onc.1206934 14576838 PMC4039039

[B7] Yusuf RZ Duan Z Lamendola DE Penson RT Seiden MV Paclitaxel resistance: molecular mechanisms and pharmacologic manipulation Curr Cancer Drug Targets 2003 3 1 1 19 10.2174/1568009033333754 12570657

[B8] Duan Z Foster R Bell DA Mahoney J Wolak K Vaidya A Hampel C Lee H Seiden MV Signal transducers and activators of transcription 3 pathway activation in drug-resistant ovarian cancer Clin Cancer Res 2006 12 17 5055 5063 10.1158/1078-0432.CCR-06-0861 16951221

[B9] Bromberg JF Wrzeszczynska MH Devgan G Zhao Y Pestell RG Albanese C Darnell JE Jr Stat3 as an oncogene Cell 1999 98 3 295 303 10.1016/S0092-8674(00)81959-5 10458605

[B10] Bromberg J Darnell JE Jr The role of STATs in transcriptional control and their impact on cellular function Oncogene 2000 19 21 2468 2473 10.1038/sj.onc.1203476 10851045

[B11] Buettner R Mora LB Jove R Activated STAT signaling in human tumors provides novel molecular targets for therapeutic intervention Clin Cancer Res 2002 8 4 945 954 11948098

[B12] Yu H Jove R The STATs of cancer--new molecular targets come of age Nat Rev Cancer 2004 4 2 97 105 10.1038/nrc1275 14964307

[B13] Nam S Buettner R Turkson J Kim D Cheng JQ Muehlbeyer S Hippe F Vatter S Merz KH Eisenbrand G Indirubin derivatives inhibit Stat3 signaling and induce apoptosis in human cancer cells Proc Natl Acad Sci USA 2005 102 17 5998 6003 10.1073/pnas.0409467102 15837920 PMC1087919

[B14] Chen CL Loy A Cen L Chan C Hsieh FC Cheng G Wu B Qualman SJ Kunisada K Yamauchi-Takihara K Signal transducer and activator of transcription 3 is involved in cell growth and survival of human rhabdomyosarcoma and osteosarcoma cells BMC Cancer 2007 7 111 10.1186/1471-2407-7-111 17598902 PMC1964761

[B15] Barre B Vigneron A Perkins N Roninson IB Gamelin E Coqueret O The STAT3 oncogene as a predictive marker of drug resistance Trends Mol Med 2007 13 1 4 11 10.1016/j.molmed.2006.11.001 17118707

[B16] Lai R Navid F Rodriguez-Galindo C Liu T Fuller CE Ganti R Dien J Dalton J Billups C Khoury JD STAT3 is activated in a subset of the Ewing sarcoma family of tumours J Pathol 2006 208 5 624 632 10.1002/path.1941 16463269

[B17] Duan Z Ames RY Ryan M Hornicek FJ Mankin H Seiden MV CDDO-Me, a synthetic triterpenoid, inhibits expression of IL-6 and Stat3 phosphorylation in multi-drug resistant ovarian cancer cells Cancer Chemother Pharmacol 2008 10.1007/s00280-008-0785-8 PMC2875930 18587580

[B18] Murray PJ The JAK-STAT signaling pathway: input and output integration J Immunol 2007 178 5 2623 2629 17312100 10.4049/jimmunol.178.5.2623

[B19] Alvarez JV Febbo PG Ramaswamy S Loda M Richardson A Frank DA Identification of a genetic signature of activated signal transducer and activator of transcription 3 in human tumors Cancer Res 2005 65 12 5054 5062 10.1158/0008-5472.CAN-04-4281 15958548

[B20] Fossey SL Liao AT McCleese JK Bear MD Lin J Li PK Kisseberth WC London CA Characterization of STAT3 activation and expression in canine and human osteosarcoma BMC Cancer 2009 9 81 10.1186/1471-2407-9-81 19284568 PMC2666757

[B21] Masuda M Suzui M Yasumatu R Nakashima T Kuratomi Y Azuma K Tomita K Komiyama S Weinstein IB Constitutive activation of signal transducers and activators of transcription 3 correlates with cyclin D1 overexpression and may provide a novel prognostic marker in head and neck squamous cell carcinoma Cancer Res 2002 62 12 3351 3355 12067972

[B22] Khoury JD Medeiros LJ Rassidakis GZ Yared MA Tsioli P Leventaki V Schmitt-Graeff A Herling M Amin HM Lai R Differential expression and clinical significance of tyrosine-phosphorylated STAT3 in ALK+ and ALK- anaplastic large cell lymphoma Clin Cancer Res 2003 9 10 Pt 1 3692 3699 14506160

[B23] Horiguchi A Oya M Shimada T Uchida A Marumo K Murai M Activation of signal transducer and activator of transcription 3 in renal cell carcinoma: a study of incidence and its association with pathological features and clinical outcome J Urol 2002 168 2 762 765 10.1016/S0022-5347(05)64741-6 12131365

[B24] Benekli M Xia Z Donohue KA Ford LA Pixley LA Baer MR Baumann H Wetzler M Constitutive activity of signal transducer and activator of transcription 3 protein in acute myeloid leukemia blasts is associated with short disease-free survival Blood 2002 99 1 252 257 10.1182/blood.V99.1.252 11756179

[B25] Dolled-Filhart M Camp RL Kowalski DP Smith BL Rimm DL Tissue microarray analysis of signal transducers and activators of transcription 3 (Stat3) and phospho-Stat3 (Tyr705) in node-negative breast cancer shows nuclear localization is associated with a better prognosis Clin Cancer Res 2003 9 2 594 600 12576423

[B26] Hsiao JR Jin YT Tsai ST Shiau AL Wu CL Su WC Constitutive activation of STAT3 and STAT5 is present in the majority of nasopharyngeal carcinoma and correlates with better prognosis Br J Cancer 2003 89 2 344 349 10.1038/sj.bjc.6601003 12865928 PMC2394270

[B27] Sheen-Chen SM Huang CC Tang RP Chou FF Eng HL Prognostic value of signal transducers and activators of transcription 3 in breast cancer Cancer Epidemiol Biomarkers Prev 2008 17 9 2286 2290 10.1158/1055-9965.EPI-08-0089 18768494

[B28] Turkson J Jove R STAT proteins: novel molecular targets for cancer drug discovery Oncogene 2000 19 56 6613 6626 10.1038/sj.onc.1204086 11426647

[B29] Duan Z Bradner JE Greenberg E Levine R Foster R Mahoney J Seiden MV SD-1029 inhibits signal transducer and activator of transcription 3 nuclear translocation Clin Cancer Res 2006 12 22 6844 6852 10.1158/1078-0432.CCR-06-1330 17121906

[B30] Duan Z Bradner J Greenberg E Mazitschek R Foster R Mahoney J Seiden MV 8-benzyl-4-oxo-8-azabicyclo[3.2.1]oct-2-ene-6,7-dicarboxylic acid (SD-1008), a novel janus kinase 2 inhibitor, increases chemotherapy sensitivity in human ovarian cancer cells Mol Pharmacol 2007 72 5 1137 1145 10.1124/mol.107.038117 17675586

[B31] Darnell JE Jr Transcription factors as targets for cancer therapy Nat Rev Cancer 2002 2 10 740 749 10.1038/nrc906 12360277

[B32] Liby KT Yore MM Sporn MB Triterpenoids and rexinoids as multifunctional agents for the prevention and treatment of cancer Nat Rev Cancer 2007 7 5 357 369 10.1038/nrc2129 17446857

[B33] Deeb D Gao X Dulchavsky SA Gautam SC CDDO-Me inhibits proliferation, induces apoptosis, down-regulates Akt, mTOR, NF-kappaB and NF-kappaB-regulated antiapoptotic and proangiogenic proteins in TRAMP prostate cancer cells J Exp Ther Oncol 2008 7 1 31 39 18472640

[B34] Ahmad R Raina D Meyer C Kufe D Triterpenoid CDDO-methyl ester inhibits the Janus-activated kinase-1 (JAK1)-->signal transducer and activator of transcription-3 (STAT3) pathway by direct inhibition of JAK1 and STAT3 Cancer Res 2008 68 8 2920 2926 10.1158/0008-5472.CAN-07-3036 18413761 PMC3092292

[B35] Shishodia S Sethi G Konopleva M Andreeff M Aggarwal BB A synthetic triterpenoid, CDDO-Me, inhibits IkappaBalpha kinase and enhances apoptosis induced by TNF and chemotherapeutic agents through down-regulation of expression of nuclear factor kappaB-regulated gene products in human leukemic cells Clin Cancer Res 2006 12 6 1828 1838 10.1158/1078-0432.CCR-05-2044 16551868

[B36] Hyer ML Croxton R Krajewska M Krajewski S Kress CL Lu M Suh N Sporn MB Cryns VL Zapata JM Synthetic triterpenoids cooperate with tumor necrosis factor-related apoptosis-inducing ligand to induce apoptosis of breast cancer cells Cancer Res 2005 65 11 4799 4808 10.1158/0008-5472.CAN-04-3319 15930300

[B37] Lapillonne H Konopleva M Tsao T Gold D McQueen T Sutherland RL Madden T Andreeff M Activation of peroxisome proliferator-activated receptor gamma by a novel synthetic triterpenoid 2-cyano-3,12-dioxooleana-1,9-dien-28-oic acid induces growth arrest and apoptosis in breast cancer cells Cancer Res 2003 63 18 5926 5939 14522919

[B38] Konopleva M Zhang W Shi YX McQueen T Tsao T Abdelrahim M Munsell MF Johansen M Yu D Madden T Synthetic triterpenoid 2-cyano-3,12-dioxooleana-1,9-dien-28-oic acid induces growth arrest in HER2-overexpressing breast cancer cells Mol Cancer Ther 2006 5 2 317 328 10.1158/1535-7163.MCT-05-0350 16505105

[B39] Suh N Wang Y Honda T Gribble GW Dmitrovsky E Hickey WF Maue RA Place AE Porter DM Spinella MJ A novel synthetic oleanane triterpenoid, 2-cyano-3,12-dioxoolean-1,9-dien-28-oic acid, with potent differentiating, antiproliferative, and anti-inflammatory activity Cancer Res 1999 59 2 336 341 9927043

[B40] Yu H Kortylewski M Pardoll D Crosstalk between cancer and immune cells: role of STAT3 in the tumour microenvironment Nat Rev Immunol 2007 7 1 41 51 10.1038/nri1995 17186030

[B41] Lourda M Trougakos IP Gonos ES Development of resistance to chemotherapeutic drugs in human osteosarcoma cell lines largely depends on up-regulation of Clusterin/Apolipoprotein J Int J Cancer 2007 120 3 611 622 10.1002/ijc.22327 17096323

[B42] Duan Z Duan Y Lamendola DE Yusuf RZ Naeem R Penson RT Seiden MV Overexpression of MAGE/GAGE genes in paclitaxel/doxorubicin-resistant human cancer cell lines Clin Cancer Res 2003 9 7 2778 2785 12855658

[B43] Chou TC Talalay P Quantitative analysis of dose-effect relationships: the combined effects of multiple drugs or enzyme inhibitors Adv Enzyme Regul 1984 22 27 55 10.1016/0065-2571(84)90007-4 6382953

[B44] Silver DL Naora H Liu J Cheng W Montell DJ Activated signal transducer and activator of transcription (STAT) 3: localization in focal adhesions and function in ovarian cancer cell motility Cancer Res 2004 64 10 3550 3558 10.1158/0008-5472.CAN-03-3959 15150111

[B45] Gritsko T Williams A Turkson J Kaneko S Bowman T Huang M Nam S Eweis I Diaz N Sullivan D Persistent activation of stat3 signaling induces survivin gene expression and confers resistance to apoptosis in human breast cancer cells Clin Cancer Res 2006 12 1 11 19 10.1158/1078-0432.CCR-04-1752 16397018

[B46] Diaz N Minton S Cox C Bowman T Gritsko T Garcia R Eweis I Wloch M Livingston S Seijo E Activation of stat3 in primary tumors from high-risk breast cancer patients is associated with elevated levels of activated SRC and survivin expression Clin Cancer Res 2006 12 1 20 28 10.1158/1078-0432.CCR-04-1749 16397019

[B47] Wesarg E Hoffarth S Wiewrodt R Kroll M Biesterfeld S Huber C Schuler M Targeting BCL-2 family proteins to overcome drug resistance in non-small cell lung cancer Int J Cancer 2007 121 11 2387 2394 10.1002/ijc.22977 17688235

[B48] Yore MM Liby KT Honda T Gribble GW Sporn MB The synthetic triterpenoid 1-[2-cyano-3,12-dioxooleana-1,9(11)-dien-28-oyl]imidazole blocks nuclear factor-kappaB activation through direct inhibition of IkappaB kinase beta Mol Cancer Ther 2006 5 12 3232 3239 10.1158/1535-7163.MCT-06-0444 17148759

